# Three-dimensional vocal fold structural change due to implant insertion in medialization laryngoplasty

**DOI:** 10.1371/journal.pone.0228464

**Published:** 2020-01-30

**Authors:** Zhaoyan Zhang, Liang Wu, Raluca Gray, Dinesh K. Chhetri

**Affiliations:** Department of Head and Neck Surgery, University of California, Los Angeles, California, United States of America; University Hospital Eriangen at Friedrich-Alexander-University Erlangen-Numberg, GERMANY

## Abstract

Glottal insufficiency due to vocal fold paralysis, paresis, or atrophy often leads to degraded voice quality. One of the primary surgical intervention procedures to treat glottal insufficiency is medialization laryngoplasty, in which an implant is inserted through a lateral window on the thyroid cartilage to medialize the vocal folds. While the goal of medialization is to modify the vocal fold structure to restore normal phonation, few studies have attempted to quantify such structural changes of the vocal folds. The goal of this study is to quantify the three-dimensional structural changes of the vocal folds due to implant insertion in medialization laryngoplasty, and evaluate its potential effect on voice production. Medialization laryngoplasty were performed in excised human larynges using implants of different stiffness. Magnetic resonance images of the larynges were obtained with and without implant insertion. The results showed that implant insertion significantly changed the original body-cover structure of the vocal folds, with the implant taking over the large space used to be occupied by the original body layer and the vocal fold being stretched into a thin layer wrapped around the implant. The medial-lateral dimension of the vocal fold was significantly reduced from about 4 mm to 1 mm, and the vocal fold was stretched in the coronal plane by about 70%. It is hypothesized that use of implants with stiffness comparable to that of the vocal folds is beneficial because the degree of medialization can be adjusted without much negative effects on phonation frequency, phonation threshold pressure, or vibration amplitude.

## Introduction

One of the primary surgical intervention procedures to treat glottal insufficiency is medialization laryngoplasty, in which an implant is inserted through a lateral window on the thyroid cartilage to medialize the vocal folds. While this procedure is effective in improving voice, the voice outcome varies significantly and the revision rate is relatively high [1, 2]. While many factors contribute to this large variability in voice outcome, the lack of a systematic and clear understanding of how implant insertion affects vocal fold geometry and stiffness and the resulting voice production is an important contributing factor [3]. In order to medialize the vocal folds, insertion of the implant would inevitably displace and deform the vocal folds and change the stiffness condition within the vocal folds. Our understanding of such changes in vocal fold geometry and stiffness is limited except for a few descriptive studies [3–5]. These studies showed that it is often difficult to anticipate the effects of implant insertion on vocal fold geometry and stiffness, particularly when the surgery planning does not take into consideration patient-specific differences in laryngeal anatomy. Currently, except for the degree of medialization that is often monitored from a superior view, changes in medial surface shape, which has been shown to play an important role in determining phonation threshold pressure, glottal closure pattern, and voice quality [6–11], are often not monitored intraoperatively. Even less is known about how such structural changes affect voice production.

The goal of this study was to quantify the three-dimensional structural changes in the vocal folds due to insertion of implants of different stiffness. It is anticipated that an improved understanding of how implant insertion affects vocal fold geometry and stiffness would provide insight into how implant insertion affects the voice outcome and identify sources of variability in voice outcomes of medialization surgery. In particular, this study focused on implants of different stiffness. Our recent study [12] showed that with stiff implants the voice outcome was very sensitive to the insertion depth, whereas implants with stiffness comparable to vocal folds provided more consistent improvement in voice outcomes across a large range of implant insertion depth. In other words, soft implants are more forgiving to surgical imprecisions in inserting and positioning implants, which is preferred considering the complexity of the medialization laryngoplasty procedure. It is hoped that this study would shed some light on the mechanisms underlying these observed differences in voice outcomes between soft and stiff implants.

A better understanding of changes in vocal fold geometry under implant insertion is also important to the development of patient-specific computational programs that may facilitate surgery planning. While recent advances in computational modeling have significantly improved computational efficiency, adaption of these models toward clinical applications has been slow, partially due to lack of experimental data of changes in vocal fold geometry and stiffness under surgical intervention. The three-dimensional experimental data presented in this study would facilitate such adaption of computational models toward clinical applications and their validations.

## Materials and methods

In this study, using magnetic resonance imaging (MRI), we first compared vocal fold geometry at the cadaveric position and under simulated activation of the lateral cricoarytenoid (LCA) muscle, which would serve as a reference of vocal fold deformation under normal vocal fold posturing. This reference deformation was then compared to changes in vocal fold geometry due to implant insertion.

Three larynges (L1: 57 years old male; L2: 82 years old female; L3: 72 years old male) were harvested from autopsy at the Department of Pathology, University of California, Los Angeles (UCLA) less than 48 hours postmortem and quick-frozen at −80°C. Written consent from organ donors’ next of kin was obtained prior to autopsy. The larynges were screened to have no laryngeal pathology or injury from intubation. One day before the MRI experiment, the larynx was allowed to thaw overnight at -4°C, and soaked in isotonic saline the morning of the experiment until completely thawed.

Larynx L1 was used to establish vocal fold deformation under normal vocal fold adduction due to LCA activation. Specifically, the right half larynx was imaged at the cadaveric resting position, whereas the left half was surgically manipulated to simulate the activation of the LCA muscle as follows: a 3–0 silk suture was placed through the muscular process of the arytenoid cartilage and both ends were advanced towards the attachment point of the LCA muscle to the cricoid cartilage. The suture was oriented lateral and parallel to the LCA muscle fibers, based on anatomical knowledge and results from our prior MRI studies [13]. One suture was then passed around the cricoid at the insertion point of the LCA muscle. LCA muscle activation was then simulated by tightening and tying the sutures to achieve full vocal fold adduction, thus also approximating the muscular process and the LCA attachment to the cricoid cartilage and shortening the LCA muscle along its length.

**Fig 1:**
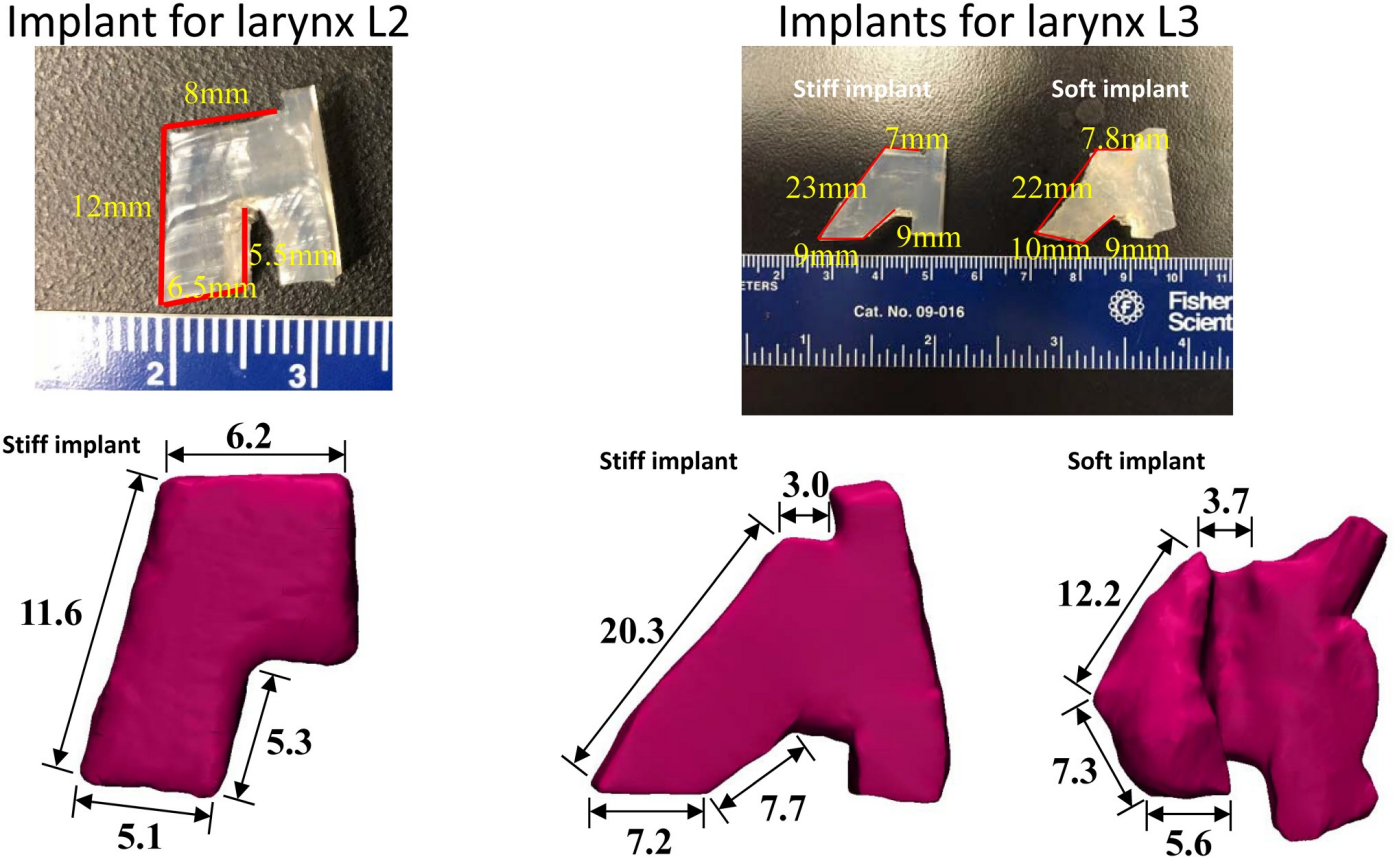
Pictures of the implants before insertion (top) and MRI reconstructions of the implants after insertion (bottom).

Larynges L2 and L3 were used to investigate changes in vocal fold geometry due to implant insertion. Specifically, L2 was used to compare vocal fold geometry at rest (left half larynx) and under insertion of a stiff implant (right half larynx), whereas L3 was used to compare vocal fold structural changes due to implants of different stiffness. Implants of two different stiffnesses were used in this study (Fig 1). The stiff implant was carved using commercially available Silastic. The soft implant was made by mixing a two-component liquid polymer solution (Ecoflex 0030; Smooth On, Inc., Easton, PA) with a silicone thinner solution, with a 1:1:2 ratio between the two components (components A and B) and the silicone thinner solution. The Young moduli of the two implants were measured using an instrumented microindentation system to be 1386 kPa and 11 kPa, respectively [12].

For implant insertion, a rectangular laryngoplasty window was created using an otologic drill. The inferior edge of the window was placed parallel to and about 2 mm from the inferior border of the thyroid cartilage. The superior edge was placed at the level of the true vocal folds, which were about half-way between the thyroid notch and the inferior border of the thyroid cartilage. The anterior border was placed 5 mm posterolateral to midline, and the posterior edge was 10 mm posterolateral to the anterior edge. The windows were approximately 10 mm × 5 mm. The cartilaginous glottis was closed by a suture placed through the two arytenoid cartilages. After insertion, the implants were further secured with sutures from outside to prevent potential displacement.

Each larynx was placed in a plastic cylindrical container (5 cm diameter) and fixed by soft foam (Mr. Clean, Cincinnati, OH). Then, the container was filled with Fomblin oil (Kurt J. Lesker Company, Livermore, CA) to prevent imaging distortions due to air-tissue interface and tissue dehydration during the long-time scan. As in the previous study [13], each larynx was scanned in a Bruker BioSpec 7 Tesla MRI (Bruker Biospin GmbH, Rheinstetten, Germany) with a 30-mm inner diameter surface coil. A rapid acquisition with relaxation enhancement (RARE) imaging sequence was selected to obtain a high-quality imaging with 3500-ms repetition time and 43-ms echo time. The spatial resolution was 100 × 100 × 100 um^3^. Based on the MRI data, the implant, cartilages (including the thyroid, cricoid, and arytenoid cartilages), intrinsic laryngeal muscles, and cover layer (the lamina propria and epithelium) were segmented using a commercial software (Simpleware, Synopsys, Inc.). Three-dimensional larynges were then reconstructed from the segmentations using Gaussian smoothing.

## Results

### Vocal fold deformation under simulated LCA activation

In this study, larynx L1 served as a reference to illustrate vocal fold deformation under normal LCA activation. LCA muscle activation was simulated by tightening the sutures that connected the muscular process and the LCA attachment to the cricoid cartilage. With proper suture placement (i.e., if the suture was placed in parallel to the LCA muscle fibers), tightening the suture would shorten the LCA muscle. Otherwise, if the suture was misaligned, tightening the suture would be more likely to bend the LCA muscle instead of shortening the LCA muscle. The three-dimensional MRI reconstruction showed that the suture manipulation shortened the LCA muscle length from 27.3 mm to 19.8 mm (a 27% shortening), and this shortening was achieved without noticeable bending of the LCA muscle, suggesting that the suture was more or less aligned with the LCA muscle fiber direction. Based on the above, it is reasonable to assume that the observed cartilage movement and vocal fold geometry changes were typical of those occurring in humans due to stimulation of the LCA muscle.

**Fig 2:**
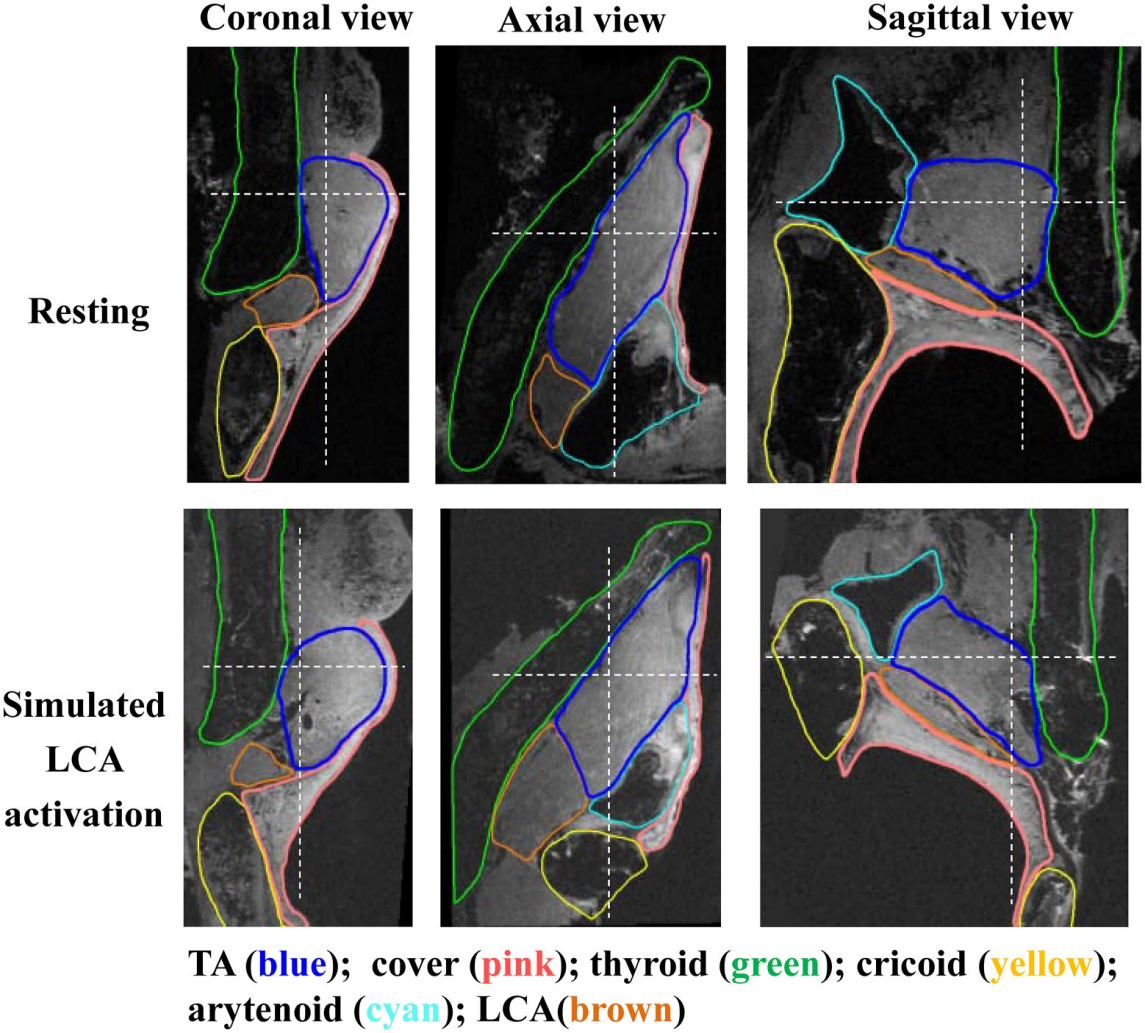
MRI images of larynx L1 (57 years old male) in three views. Top: the left half larynx at the resting position. Bottom: the right half under simulated activation of the lateral cricoarytenoid muscle. The dashed white lines in each view indicate the cut planes from which the other two views were generated.

Fig 2 shows the MRI images of larynx L1 in coronal, axial, and sagittal views with superimposed segmentations of the thyroarytenoid (TA) muscle, vocal fold cover, and cartilages. The simulated LCA activation caused a forward, medial, and downward rotation of the arytenoid cartilage, which medialized the vocal folds, particularly in the posterior membranous glottis. In the coronal plane, LCA activation induced a medial and downward rotation, which led to a more rectangular medial surface in contrast to a more convergent medial surface shape at the resting position. The vertical thickness of the rectangular portion of the medial surface, measured as the vertical span of the most medial portion (1 pixel or about 0.1 mm depth in the medial-lateral direction) of the vocal folds, almost doubled (Table 1). Such changes in the medial surface shape have been shown to lower the phonation threshold pressure [9–11] and prolong glottal closure during phonation [6–8]. There was no noticeable change in the medial-lateral depth of the TA muscle.

**Table 1:** MRI measurements of vocal fold medial surface vertical thickness and the medial-lateral depth of the TA and cover layer at three longitudinal locations (anterior, middle, and posterior).

	medial surface vertical thickness (mm)			cover/TA medial-lateral depth (mm)		
	anterior	middle	posterior	anterior	middle	posterior
L1, resting	1.11	1.29	1.10	0.67/5.44	0.52/6.36	0.55/6.17
L1, simulated LCA	2.15	2.73	2.38	0.88/5.85	0.77/6.63	0.80/7.59
L2, resting	1.91	2.08	2.66	0.55/3.18	0.54/4.32	0.67/4.81
L2, stiff implant	4.56	4.02	3.28	0.43/0.41	0.52/1.11	0.61/1.70
L3, soft implant	4.20	3.65	2.54	0.51/1.33	0.60/1.36	0.50/1.35
L3, stiff implant	5.24	3.73	2.04	0.47/2.43	0.35/2.46	0.49/2.56

### Vocal fold deformation due to implant insertion

**Fig 3:**
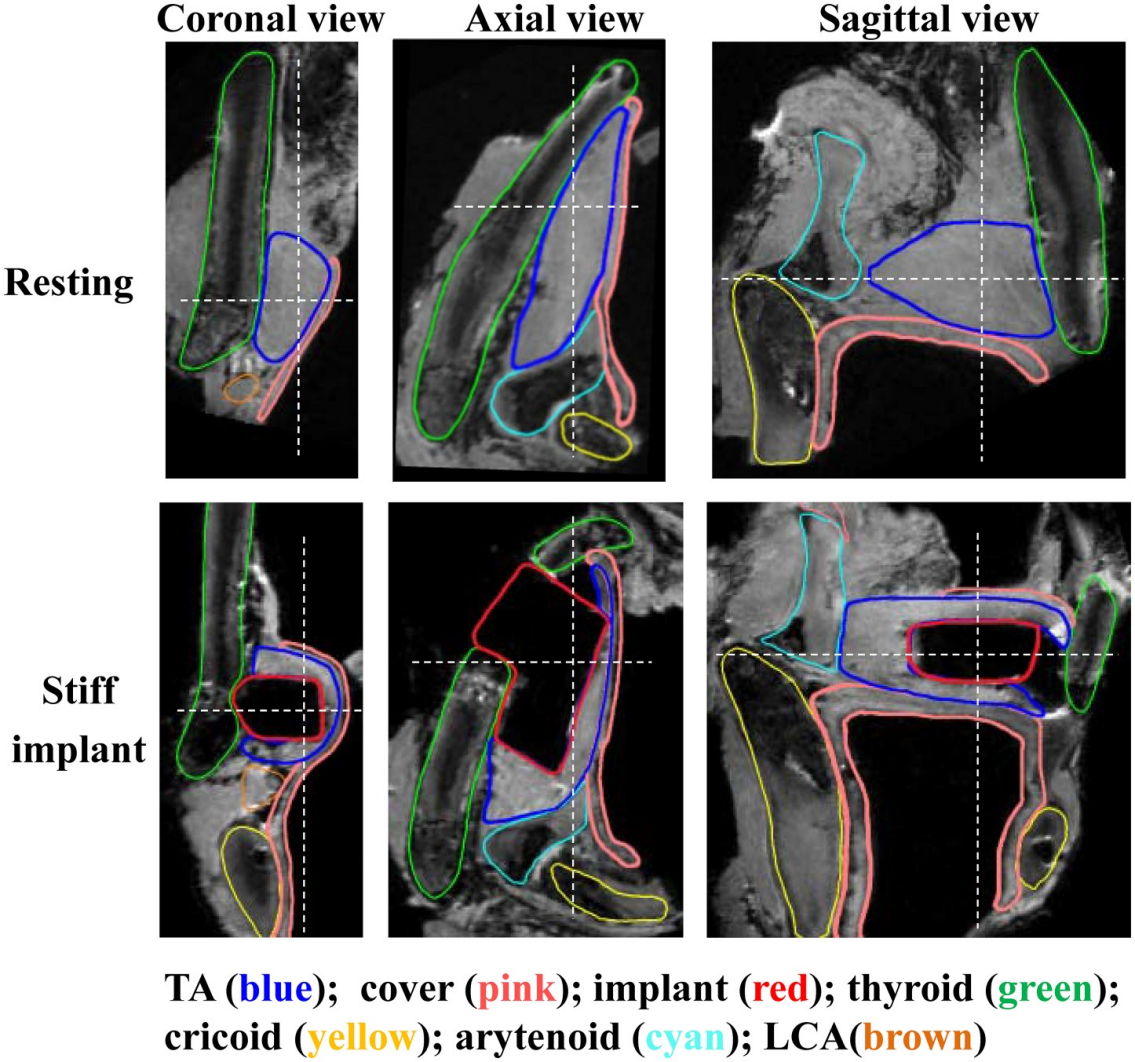
MRI images of larynx L2 (82 years old female) in three views. Top: the left half larynx at the resting position. Bottom: the right half with insertion of a stiff implant. The dashed white lines in each view indicate the cut planes from which the other two views were generated.

Fig 3 shows the MRI images of larynx L2. A stiff (Silastic) implant was inserted into the right half while no implants were used in the left half. Insertion of the implant did medialize at least the anterior portion of the membranous vocal folds from a superior view. Unlike that in larynx L1 in which medialization was achieved through arytenoid rotation, medialization in larynx L2 was achieved without much motion of the arytenoid cartilage. In the coronal view, implant insertion changed the medial surface to be less convergent and more rectangular than that at rest. Compared with larynx L1 under simulated LCA activation, implant insertion significantly increased the vertical thickness of the medial surface (from 2 mm to about 4 mm).

One major structural change with implant insertion was that the TA muscle was severely compressed along the medial-lateral direction and stretched into a thin layer wrapped around the implant. The medial-lateral depth of the TA muscle in the medial surface region was significantly reduced from about 4 mm to about 1mm (Table 1), a 75% reduction. The depth of the TA muscle was almost zero at an anterior location at which the anterior corner of the implant pressed against the TA muscle (Fig 3). This medial-lateral compression was accompanied by an approximately 70% elongation of the TA muscle vertically in the coronal plane, from 8.99 mm to 15.4 mm (Table 2). Due to material nonlinearity, this stretching was likely to significantly increase both the transverse stiffness and tension within the vocal folds. Note that while the TA muscle may appear to be compressed by the implant in Fig 3, comparison in the TA volume between the left and right halves showed minimal volume changes due to implant insertion (Table 2), although the stretching did significantly increase the surface area of the TA muscle.

**Table 2:** MRI measurements of TA muscle volume, TA surface area, vertical span of the TA muscle before implant insertion, and TA contour length after implant insertion.

	TA volume (mm3)	TA surface area (mm2)	TA vertical span in coronal plane before implant (mm)	TA contour length in coronal plane after implant (mm)
L1, resting	699	566	11.37	n/a
L1, simulated LCA	631	492	10.78	n/a
L2, resting	386	375	8.99	n/a
L2, stiff implant	376	568	n/a	15.4
L3, soft implant	579	837	n/a	16.8
L3, stiff implant	556	671	n/a	14.3

Fig 3 also shows some gaps between the implant (e.g., the anterior surface of the implant) and the vocal fold tissue in the sagittal view, probably due to the large stiffness difference between the implant and soft tissue which may have prevented the implant to better mold with the soft tissue being displaced.

**Fig 4:**
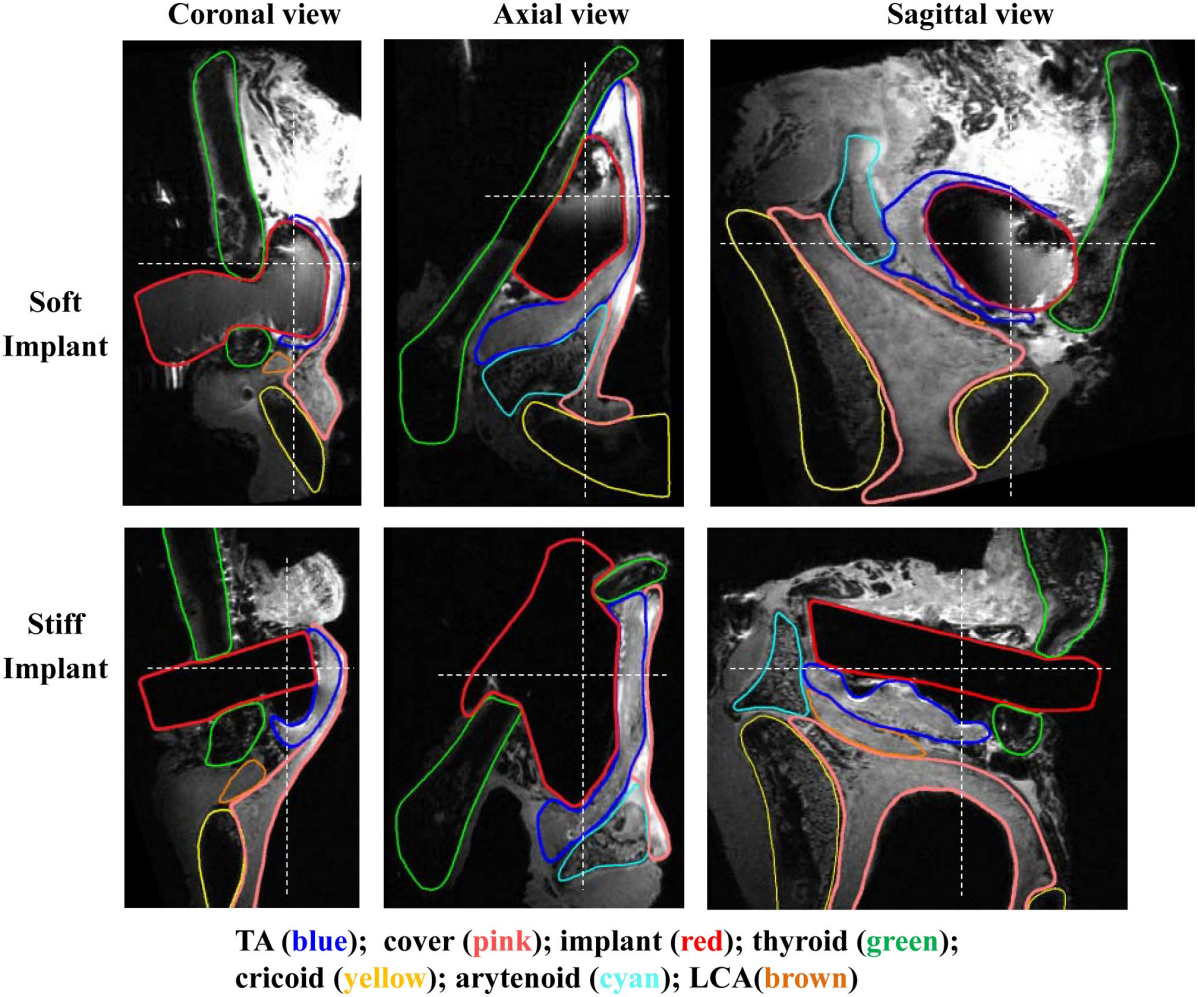
MRI images of larynx L3 (72 years old male) in three views. Top: the left half larynx with insertion of a soft implant. Bottom: the right half with insertion of a stiff implant. The dashed white lines in each view indicate the cut planes from which the other two views were generated.

Fig 4 compares vocal fold deformation in larynx L3 with the soft implant inserted on the left and the stiff implant on the right. Similar vocal fold compression and stretching can be observed in both implants. Note that in the case of the stiff implant, while the original goal was to aim the implant toward the vocal fold medial surface and this appeared so from the superior view, the implant was actually found positioned slightly above the plane of the vocal folds. Consequently, the degree of vocal fold compression (or reduction in medial-lateral depth, Table 1) was not as much as in larynx L2.

The stiff implant also led to tears in the vocal fold tissue of larynx L3, as shown in Fig 4. In the sagittal view, the tissue edge became irregular in the posterior portion right underneath the implant. There appeared to be also some tear in the anterior vocal fold above the implant. Since there were much less tears on the other fold in the same larynx, they were less likely due to the damages in the freezing or thawing process. Such tears likely occurred during the implant insertion process in which a large shear force may be generated due to the large stiffness mismatch between the implant and the soft tissue. The coronal view in Fig 4 also shows a gap between the undersize of the implant and the TA muscle.

Compared to the case of the stiff implant in which the implant was able to maintain its original shape, the soft implant experienced considerable deformation in the implant insertion process. In general, the soft implant was able to mold better with the vocal fold tissue, as shown in the sagittal view which shows the soft implant was deformed from its original shape and was surrounded closely by vocal fold tissue. There were also not many tears in the vocal fold tissue or gaps between the soft implant and the vocal folds. However, this implant deformation also means it would be difficult to anticipate how implant insertion would shape the vocal fold and its medial surface.

## Discussion and conclusions

Hirano proposed that the vocal folds can be functionally divided into a body and a cover layer, with the body layer consisting of the TA muscle and the deep layer of the lamina propria, and the cover layer consisting of the superficial and intermediate lamina propria and the epithelium [14]. Our study showed that, with implant insertion, the implant took over the space used to be occupied by the TA muscle, whereas the TA muscle was compressed and stretched into a thin layer around the implant and became much less dominant structurally. The larger the implant insertion depth, the more compressed the TA muscle is, and the more dominant the implant will be structurally. Our study showed that in order to medialize the vocal folds, at least from a superior view, the insertion depth required often resulted in the implant occupying about 75% of the medial-lateral depth of the vocal fold-implant system. With the implant occupying a large portion of the vocal fold-implant system, its mechanical properties and geometry are expected to have a significant effect on voice production in implanted larynges, especially with implants that are much stiffer than the vocal folds, e.g., Silastic as in this study and a popular choice in medialization laryngoplasty surgery.

Although the implant-vocal fold configuration is structurally similar to the original body-cover structure, implant insertion does not provide or restore in a paralyzed larynx the regulatory function of the TA muscle in regulating phonation frequency and voice types, due to difficulties in regulating implant stiffness after insertion, an inherent limitation of current passive thyroplasty implants. On the other hand, in conditions where muscular stimulation is not completely lost (e.g., vocal fold atrophy or paresis), the regulatory function of the TA muscle, now compressed and stretched into a thin layer, is expected to be affected by implant insertion, which should be investigated in future studies.

The effect of the structural changes due to implant insertion on voice production was not investigated in this study. However, findings from previous studies can shed some light on the potential effects of implant stiffness. It has been shown that increasing the body layer stiffness increases phonation frequency and phonation threshold pressure, and decreases vibration amplitude [15, 16]. In the extreme case when the implant (e.g., Silastic as in this study) is much stiffer than the vocal folds, the increase in phonation frequency and phonation threshold pressure may be too high to be desirable in bilateral implantation. A Silastic implant coupled with a thin compressed vocal folds is also likely to reduce vibration amplitude, and may even suppress vibration at locations where the vocal fold tissue is extremely thin (e.g., Fig 3, axial plane where the anterior corner of the implant pressed against the medial surface). Such negative effects have been reported in our recent study [12]. It appears that stiff implants may not be ideal for larynges with already reduced vocal fold volume (e.g., presbylarynges or atrophied larynges), in which it may be difficult to obtain good closure with a very thin vocal folds on top of a stiff implant.

In unilateral implantation, a very stiff implant would make it difficult for the contralateral fold to match its stiffness, and may result in large left-right stiffness mismatch and asymmetric vocal fold vibration, which may further degrade voice quality [17, 18]. However, contralateral fold compensation may also reduce some of the negative effects of stiff implants. For example, the increase in phonation frequency due to stiff implant insertion is expected to be smaller in unilateral implantation than that in bilateral implantation, because phonation frequency is determined by the stiffness conditions of both the contralateral fold and the implanted fold [17].

One disadvantage of stiff implants is that these negative effects (increased phonation threshold pressure, increased phonation frequency, suppressed vibration amplitude) are tied to the degree of medialization. The only way to mitigate these negative effects of stiff implant is to reduce the geometric proportion of the implant by reducing implant insertion depth, which would likely lead to insufficient medialization. As a result, when stiff implants are used, the voice outcome is likely to be sensitive to insertion depths, particularly in bilateral implantation, as observed in our recent study Zhang et al. [12]. Zhang et al. showed that full insertion of stiff implants led to significant improvement in voice acoustics but at a very high phonation frequency and requiring high subglottal pressure. Reducing the insertion depth reduced the phonation frequency but at the cost of reduced voice improvement. Such high sensitivity is not desirable as it requires high surgical precision in implant insertion, which is difficult to achieve intraoperatively.

Zhang et al. also showed that soft implants do not have such sensitivity to insertion depth [12]. This is probably because with implants of comparable stiffness to that of vocal folds, changing insertion depth will not significantly change the overall stiffness of the vocal fold-implant system. Thus, the degree of medialization can be adjusted without much influence on the phonation frequency, phonation threshold pressure, or vibration amplitude. This is particularly important when medializing larynges with atrophy, in which a large implant size is required to sufficiently medialize the vocal folds, and soft implants are likely to produce better improvement in the voice outcome than stiff implants.

In this study insertion of either soft or stiff implants modified the medial surface shape from convergent to more rectangular with noticeable increases in vertical thickness of the medial surface. Note that such modifications in medial surface shape were achieved even in larynx L3 where the stiff implant was directed slightly upwards. It appears that the upward and medial displacement of the implants may have applied a downward and medial force toward the vocal folds, which caused the inferior portion of the medial surface to bulge medially and squared up the medial surface. Considering the important effect of medial surface shape on voice production, a better understanding of how implants interact with vocal folds to affect the medial surface shape, particularly when implants of comparable stiffness are used, would help better plan medialization surgery.

The significant amount of compression and stretching (about 70%) of the vocal folds is also likely to change the stiffness and tension conditions in the vocal folds. Specifically, compression is likely to introduce negative tension along the medial-lateral direction in the vocal folds. Due to material nonlinearity, stretching of the vocal folds around the implant would significantly increase vocal fold stiffness in the transverse plane. This may explain the stiffness increase reported in a recent indentation study [19]. Previous studies have shown that increasing transverse stiffness may increase phonation threshold pressure and reduce the vertical phase difference and thus the closed quotient of vocal fold vibration [20], which may be undesirable, but may also suppress vocal instabilities [21].

Our study also shows that implant insertion may create separations or gaps between the vocal fold and the implant or the thyroid cartilage. It is unclear whether such separations or gaps would occur in vivo, or if they occur, whether they would disappear with prolonged voice use. Mechanically, such separations or gaps weakens the lateral support that would otherwise be provided by laryngeal cartilages or surrounding tissue, which may result in larger vibration amplitude but may also allow vocal folds to be more easily displaced laterally by the intraglottal pressure, thus reducing the degree of glottal closure. Their potential effects need to be better understood in future studies.

Although only three larynges were examined in this study, the main conclusions (i.e., implant insertion changes the body-cover structure of the vocal fold and the potential effects of implant stiffness due to the large volume proportion occupied by the implant) are likely to remain the same in other larynges. One important limitation of our study, however, was that simultaneous phonation experiments were not conducted so that the observed structural changes cannot be directly related to their vibrational and acoustic outcomes, which should be investigated in future studies. Also, more systematic computational modeling studies should be conducted to better understand the effects of the observed structural changes on voice production toward optimizing implant selection and positioning.
